# Thin Endometrium Is Also Associated With Lower Clinical Pregnancy Rate in Unstimulated Menstrual Cycles: A Study Based on Natural Cycle IVF

**DOI:** 10.3389/fendo.2018.00776

**Published:** 2018-12-20

**Authors:** Michael von Wolff, Monika Fäh, Marie Roumet, Vera Mitter, Petra Stute, Georg Griesinger, Alexandra Kohl Schwartz

**Affiliations:** ^1^Division of Gynaecological Endocrinology and Reproductive Medicine, University Women's Hospital, Inselspital University Hospital, Bern, Switzerland; ^2^CTU Bern, Institute of Social and Preventive Medicine, University of Bern, Bern, Switzerland; ^3^Division of Gynaecological Endocrinology and Reproductive Medicine, University Women's Hospital, University Hospital Schleswig-Holstein, Lübeck, Germany

**Keywords:** endometrium, pregnancy rate, live birth rate, natural cycle IVF, spontaneous cylce

## Abstract

**Introduction:** Does the endometrial thickness (EMT) at the time of follicle aspiration correlate with the pregnancy rate in unstimulated menstrual cycles?

**Materials and Methods:** This is a retrospective, observational single center study.105 women with regular menstrual cycles undergoing their first NC-IVF cycle with an embryo transfer were analyzed. Clinical pregnancy and live birth rates were calculated and data were adjusted for women's age, cycle day of follicle aspiration and body mass index (BMI).

**Results:** Age of participants was 35.0 y [32.0; 37.0]. Follicle aspiration was performed on day 14.0 [12.0; 15.0] of the cycle. Total clinical pregnancy rate was 24.8% and live birth rate 15.2% per transfer. Pregnancy rate in women with endometrial thickness ≤7 mm (*n* = 27) was 7.4 and 30.8% in women >7 mm (*n* = 78) (OR 5.56, 1.22–25.36) (*P* = 0.03). Live birth rates were not significantly different. Quadratic regression analysis revealed lower pregnancy rates in women with thin (around <8 mm) as well as with thick (around >11 mm) endometria. *P*-value after crude quadratic analysis was 0.028 and after adjustment for age, day of aspiration and BMI was 0.039. Significance was not reached for live birth rates.

**Conclusion:** Thin endometrium should also be considered as an independent negative prognostic factor for achieving pregnancy in women without ovarian stimulation.

## Introduction

The importance of the endometrium for the development and maintenance of pregnancy is clearly proven. However, it is unclear which endometrial factors are of relevance (1). Histological examination in couples who wish to conceive makes little sense as a biopsy would be necessary. In transvaginal ultrasound evaluation endometrial thickness, the echo pattern and endometrial perfusion are evaluated (2). Ultrasound analysis of endometrial thickness (EMT) is most commonly performed, as this is the easiest and best reproducible technique.

The significance of the endometrial thickness has been investigated in numerous studies and in meta-analyses. The investigations are essentially limited to *in vitro* fertilization treatments (IVF) with high-dose stimulation therapy and intrauterine insemination treatment (IUI) with different ovarian stimulation regimes.

In IVF treatments, a thin endometrium is associated with lower pregnancy rates. The clinical pregnancy rate is related to a lower chance of pregnancy if endometrial thickness is ≤7 mm (OR 0.42, 95% CI: 0.27, 0.67) (3). The data regarding a thick endometrium is not so clear. A previous study described reduced pregnancy rates in women with endometrium >14 mm (4), whereas other studies did not find decreased or found even increased pregnancy rates (5–7).

In women undergoing IUI with low dose stimulation, such a relationship does not appear to exist. In a recent meta-analysis with IUI treatments combined with a gonadotrophin, clomifene citrate, or aromatase inhibitor stimulation, there was no evidence of a difference in EMT between women who conceived and women who did not conceive (MDrandom: 0.51, 95% CI: −0.05, 1.07) (8).

Because ovarian stimulation treatments were used, the results of these studies cannot be transferred to the unstimulated situation. As a result, the study findings have only limited use in a fertility work-up for infertility to assess the relevance of the endometrium as a cause of sterility.

Based on this, we investigated the pregnancy rate as a function of the endometrial thickness using the Natural Cycle IVF (NC-IVF) model, in which no ovarian stimulation except ovulation induction with human chorionic gonadotropin, hCG, and luteal phase progesterone supplementation was administered.

Since strict inclusion and exclusion criteria such as the transfer of only one embryo were defined and thus numerous confounders on the pregnancy rate could be excluded, this may be the first study which also allows a cautious estimate of the importance of endometrial thickness for a pregnancy event in a spontaneous cycle.

## Methods

### Study Population and Participants

The retrospective, observational single center study was performed between 2011 and 2016. A total of 225 women, 18–42 years of age with regular menstrual cycles (24–32 days) and basal FSH concentrations <10 IU/L undergoing their first IVF cycle treatment with transfer of a single embryo were screened. Women had been offered both, NC-IVF and conventional IVF but decided themselves which therapy they preferred. Women without a transfer, with endometriosis >rAFS II° (revised American Fertility Society) (as diagnosed by laparoscopy or clinical and ultrasound analysis), with fibroids as diagnosed by ultrasound or in case ultrasound was not conclusive by hysteroscopy and with sperm collection by testicular sperm extraction (TESE) were excluded.

NC-IVF patients were monitored using ultrasound and analysis of luteinizing hormone (LH) and E_2_ concentrations. When the follicle diameter reached at least 18 mm and the E_2_-concentration was expected to be ≥800 pmol/L, 5000 IU of hCG (Pregnyl®, MSD Merck Sharp & Dohme GmbH, Lucerne, Switzerland) were administered and patients were scheduled 36 h later for oocyte retrieval. EMT was measured at the time of oocyte aspiration by different physicians and different ultrasound machines. Endometrium thickness was measured in mm without decimal numbers in our clinical routine as intraindividual and interindividual variations did not justify a more precise measurement. Follicles were aspirated without anesthesia and without analgesia using 19G single lumen needles (220 mmHg) as described elsewhere (9). After aspiration, follicles were flushed and aspirated 3 times each using 2–5ml flushing medium with heparin (SynVitro^®^ Flush, Origio, Berlin, Germany). The flushing volume was adapted according to the size of the follicles. Fertilization was achieved by standard ICSI in all cases. Embryos were transferred on day 2 or 3 after aspiration as long term culture was not required with only one embryo. Women received luteal phase support with vaginal micronized progesterone. EMT at the time of follicle aspiration as well as biochemical and clinical (defined as ultrasound detection of an amniotic sac) pregnancy rates and live birth rate were analyzed per embryo transfer.

The study was carried out in accordance with the recommendations of the local ethical committee of the IRB Internal Review Board, Inselspital Bern on October 12th 2012 (IRB 12–223). All subjects gave informed written consent in accordance with the Declaration of Helsinki.

### Statistical Analysis

Endometrial thickness was first considered as a categorical variable and therefore women were divided into two endometrial thickness groups (≤7 mm vs. >7 mm). Patients' baseline characteristics were compared for quantitative variables by using the *t*-test, or if normality assumption was not satisfied, by non-parametric Wilcoxon-test. For qualitative variables (cause of infertility), the Chi-square test was used or by Fisher's exact test when the sample size was small.

Clinical pregnancy and life birth rate where compared using a logistic regression. For each outcome, we first assessed the crude (unadjusted) association between EMT categories and the outcome. We then adjusted the model for potential confounders by including women's age, day of follicle aspiration and BMI in the model. The cause of the infertility was not considered as we had been shown in another, not yet published, study, that the cause of infertility is not a prognostic factor in NC-IVF. Endometrial thickness was then considered as a continuous variable and its effect on pregnancy and on live birth was further analyzed using logistic regression. For each outcome, we first assessed the crude (unadjusted) and adjusted association between EMT and the outcome, using endometrial thickness as a linear term. Then, we examined the linearity of the effect of EMT on the outcomes by fitting a crude and adjusted quadratic regression model and by testing whether the addition of a quadratic term significantly increased the fit of the model. Models were compared using likelihood ratio tests. *P*-value and the confidence interval of models parameters were estimated using the normal approximation.

## Results

Two hundred and twenty five women undergoing a NC-IVF cycle were identified. One hundred and eleven women (49.3%) were excluded due to missing transfer (premature ovulation 14%, aspirations without oocyte 15%, oocytes without fertilization or arrested embryo growth 16%), 5 women (2.2%) due to endometriosis, and 4 women (1.8%) due to TESE, resulting in 105 women to be included in the analysis. Cycles and transfers were not canceled due to thin endometrium. The basic characteristics of these women are shown in Table 1. Age of participants was 35.0 y [32.0; 37.0], cycle day of follicle aspiration was 14.0 [12.0; 15.0] and duration of infertility was 3.00 y [2.00; 4.00]. Infertility factors were severe male factor (sperm < 5 Mill/ml) (*n* = 26, 24.8%), moderate and mild male factor (sperm 5- < 15 Mill/ml or total motility <40%) (*n* = 24, 22.9%), tubal factor (peritubal adhesions, blockage of one or both fallopian tubes), endometriosis rAFS I-II° and mixed factors (*n* = 22, 21.0%), and idiopathic infertility (*n* = 33, 31.4%).

**Table 1:** Baseline characteristics of all analyzed patients (*n* = 105) and of women with endometrial thickness ≤7 mm (*n* = 27) vs. >7 mm (*n* = 78) (data are shown as median and upper and lower quartile ranges).

	**Total (*n =* 105)**	**EMT ≤7 mm (*n =* 27)**	**EMT >7 mm (*n =* 78)**	***P*-value**
Age at time of aspiration (years)	35.0 (32.0; 37.0)	37.0 (33.5; 39.5)	34.0 (32.0; 36.0)	0.06
Cycle day of follicle aspiration	14.0 (12.0; 15.0)	14.0 (13.0; 15.0)	14.0 (12.0; 15.0)	0.46
Infertility since (years)	3.00 (2.0; 4.0)	3.0 (2.0; 3.0)	3.0 (2.0; 4.0)	0.41
Body mass index, BMI	21.3 (20.0; 23.4)	20.8 (19.2; 22.3)	21.6 (20.2; 23.5)	0.13
Cause of infertility (n, %)				0.98
Severe male factor	26(24.8%)	7(25.9%)	19(24.4%)	
moderate/mild male factor	24(22.9%)	6(22.2%)	18(23.1%)	
Tubal factor, endometriosis rAFS I-II° and mixed factors	22(21.0%)	5(18.5%)	17(21.8%)	
Idiopathic	33(31.4%)	9(33.3%)	24(30.8%)	
Thickness of the endometrium(mm)	8.0 (7.0; 10.0)	7.0 (7.0; 7.0)	9.0 (8.0; 10.0)	Not applicable

**Figure 1:**
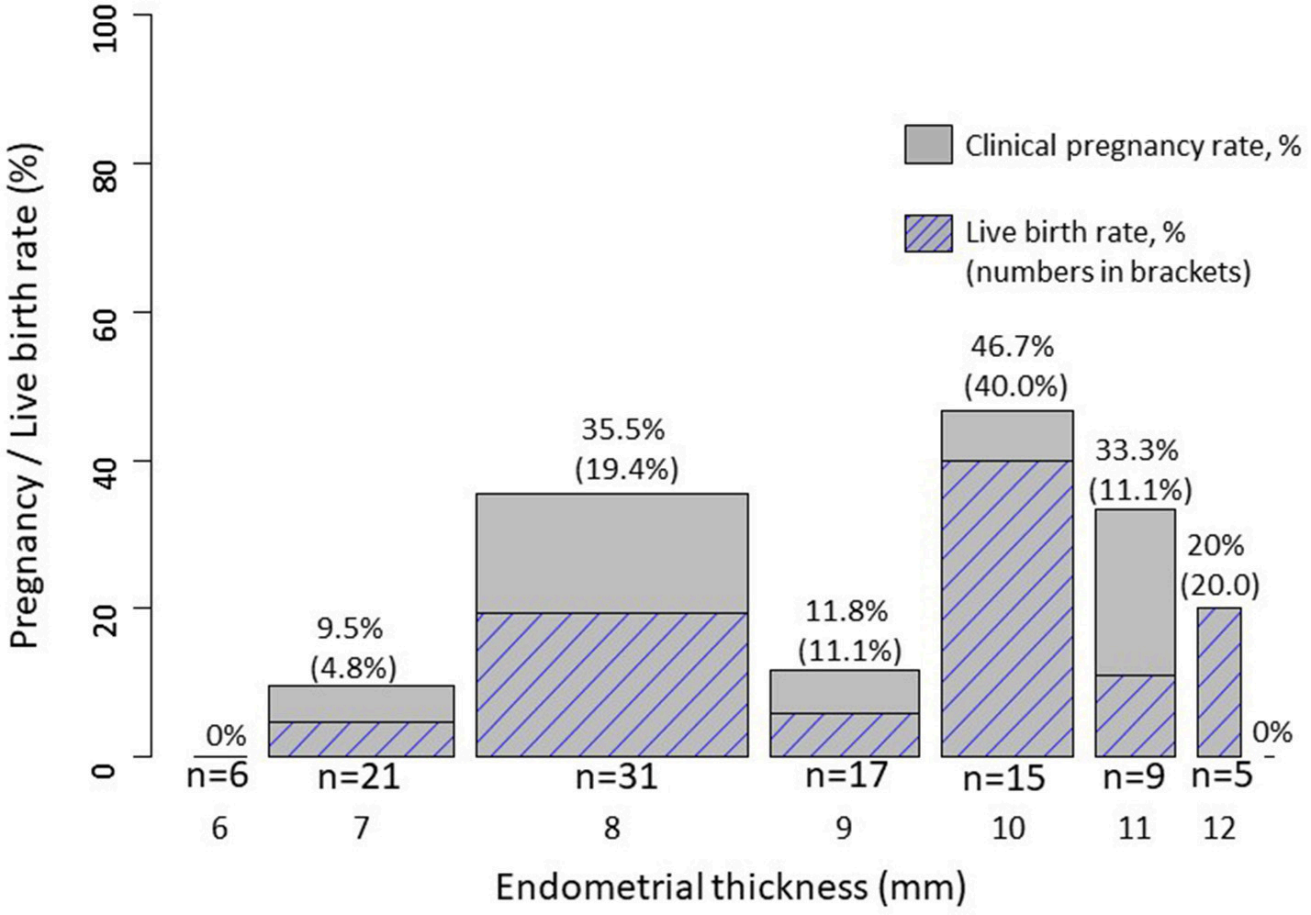
Clinical pregnancy (not hatched) and live birth (hatched) rates as a function of endometrial thickness. The bar width is proportional to the number of women in each category.

**Figure 2:**
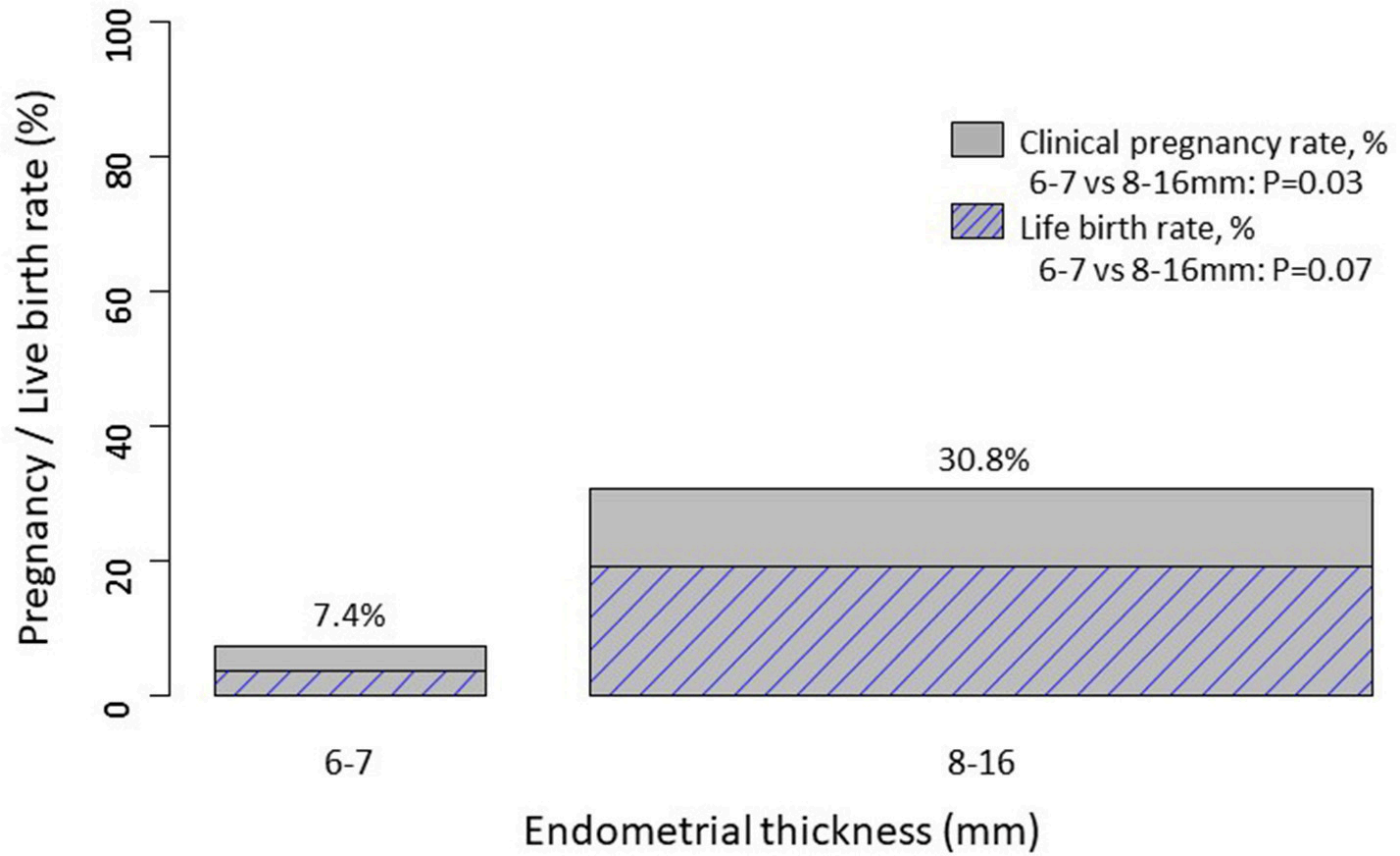
Clinical pregnancy rate (not hatched) and live birth (hatched) rates in women with endometrial thickness ≤7 mm vs. >7 mm. The bar width is proportional to the number of women in each category.

Overall AMH concentrations were 12.0 pmol/l [6.0; 22.0]. Clinical pregnancy rate and live birth rate as a function of endometrial thickness are shown in Figures 1, 2. Endometrial thickness was 6 mm in 6 women, 7 mm in 21, 8 mm in 31, 9 mm in 17, 10 mm in 15, 11 mm in 9, 12 mm in 5, and 16 mm in 1 woman.

The pregnancy rate was firstly compared in women with endometrial thickness ≤7 mm (*n* = 27) vs. >7 mm (*n* = 78). The groups did not differ regarding women's age, cycle day of aspiration, duration of infertility, BMI and cause of infertility (Table 1). Clinical pregnancy rates in women with endometrial thickness ≤7 mm (*n* = 27) vs. >7 mm (*n* = 78) were 7.4 and 30.8%, respectively.

Crude and adjusted logistic regression, adjusted for women's age, day of follicle aspiration and BMI, showed an increased odds of pregnancy in patients with increased endometrial thickness >7 mm (crude OR 5.56, 95% CI: 1.22–25.36, *p* = 0.027; adjusted OR 5.50, 95% CI: 1.14–26.62, *p* = 0.034). Likewise, the odds of live birth was also increased in patients with increased endometrial thickness, however, associations were not significant (crude OR 6.19, 95% CI: 0.78–49.27, *p* = 0.085; adjusted OR 5.98, 95% CI: 0.74–48.56, *p* = 0.094).

We also used endometrial thickness as a continuous variable in logistic models. There is a not significant trend of a linear relationship between continuous endometrial thickness and pregnancy [crude OR (per log (mm)): 6.63, 95% CI: 0.61–71.99, *P* = 0.12]; adjusted OR [per log (mm)): 4.52, 95% CI: 0.38–53.14, *P* = 0.23]. Likewise, there is a non-significant trend of a linear, non-significant relationship between continuous endometrial thickness and live birth [crude OR (per log (mm)): 8.99, 95% CI: 0.54–150.06, *P* = 0.13]; adjusted OR [per log (mm)): 8.02, 95% CI: 0.41–157.41, *P* = 0.17]. However, the model fit is better when modeling a quadratic relationship instead of a linear relationship between endometrial thickness and clinical pregnancy (*p*-value from a model comparison = 0.03). The crude and adjusted quadratic models indicate a decreased odds of pregnancy for thinner (around <8 mm, *n* = 27) but also for very thick (around >11 mm, *n* = 5) endometrium (*p*-value for quadratic relationship: crude *p* = 0.028; adjusted *p* = 0.039).

Likewise, there was also a trend of a better model fit when modeling a quadratic relationship between endometrial thickness and live birth (*p*-value from a model comparison = 0.08). The crude and adjusted quadratic models indicate a decreased odds of pregnancy for thinner but also for very thick endometrium (*p*-value for quadratic relationship: crude *p* = 0.066; adjusted *p* = 0.093).

## Discussion

### Main Findings

This study described for the first time the association of pregnancy rates with endometrial thickness in unstimulated menstrual cycles with fresh embryo transfers. The evaluation was adjusted for the main factors that could influence the chance of pregnancy (age) (10) and the EMT (day of aspiration and BMI) (11).

### Strengths and Limitations

To minimize the influence of possible influencing variables, the investigation was carried out using 105 NC-IVF cycles, in which–as in almost all NC-IVF cycles–only one embryo was transferred. Different numbers of embryos would not have allowed a comparison of pregnancy rates. However, it needs to be noted that first we performed a retrospective analysis and second that the strict inclusion and exclusion criteria resulted in a limited number of participants. This might be a reason why significance was only reached for pregnancy but not for live birth rate.

Endometrium thickness was analyzed by several physicians using different ultrasound machines. Therefore, and due to the intra und interindividual variations of endometrial measurements, EMT was analyzed without decimal numbers, which could have affected the precision of the analysis.

We defined the ultrasound detection of an amniotic sac as a clinical pregnancy, which might explain the high miscarriage rate. However, the miscarriage rate could not be allocated to a specific endometrial thickness.

### Interpretation

In all studies published to date, the association of endometrial thickness with pregnancy rate was performed only with high-dose IVF stimulations, cryopreserved embryos (12) or low-dose IUI stimulations. The IVF studies suggested an increase in the pregnancy rate with an endometrium >7 mm (3), whereas the IUI studies failed to demonstrate such a relationship (8). Our study confirmed the reduced pregnancy rate in gonadotrophin-stimulated IVF therapies with an endometrial thickness of ≤7 mm. However, an increase in the pregnancy rate in a particularly thick endometrium of >11 mm (6), >13 mm (5), or >14 mm (7), as demonstrated with gonadotrophin-stimulated IVF therapies, could not be confirmed. In contrast, we even observed a tendency to lower pregnancy rates in women with particularly thick endometrium. In IUI treatments with low dose gonadotrophin stimulation, neither an increased nor a reduced pregnancy rate (8) was found.

The reason for the reduced pregnancy rates in patients undergoing gonadotrophin-stimulated IVF therapies with an endometrial thickness ≤ 7 mm compared to an endometrial thickness >7 mm is unclear. It has been speculated that basal layer endometrial oxygen concentrations are increased in patients with a thin endometrium, which might be detrimental for embryo implantation (13). It was further speculated that embryos developing *in vitro* are especially susceptible to this higher oxygen exposure (8).

In hormone-stimulated IUI treatments, there is no significant correlation between the endometrial thickness and the pregnancy rate (8). As a possible reason for this, it was discussed that embryos develop more robustly *in vivo* and are less susceptible to high oxygen exposure (8). However, this explanation is purely hypothetical. Therefore, it could be speculated that in hormone-stimulated IUI treatments a thin endometrium is associated with lower pregnancy rates, but that this association could not be detected. In the meta-analysis by Weiss et al. (8), the primary analysis showed a significantly thinner endometrium in women who did not conceive (MD: 0.48, 95% CI: 0.18, 0.77). The significance was only lost when the calculation was performed using a random effects model (MD random: 0.51, 95% CI: −0.05, 1.07) which was chosen due to the heterogeneity of studies. This raises the question of whether a thin endometrium is also associated with lower pregnancy rates in stimulated IUI treatments; however, this association could not be detected due to the heterogeneity across the studies.

Pregnancy rates have also been studied in modified natural cycles with frozen-thawed embryo transfers (12). Mean endometrial thickness did not differ between patients achieving ongoing pregnancy and those who did not. However, the pregnancy rates in women with endometrium of <7 mm (*n* = 41) was only 9.8% whereas in women with an endometrium of ≥7 mm it was 21.0% (12). Even though the differences were not statistically different, these data support the hypothesis, that pregnancy rates are lower in women with thin endometrium, even in unstimulated cycles.

Lower pregnancy rates with a thin endometrium in unstimulated cycles are unlikely to be biologically plausible. It is unlikely that a tendency toward a thin endometrium could be inherited if it significantly affected fertility. There are, of course, numerous factors that lead to a thin endometrium or which are associated with a thin endometrium and lower the chances of pregnancy. The most relevant factors are multiple curettages (14) and exposure of the uterus to radiation (15). However, these factors are either iatrogenic or due to an acquired pathology and therefore cannot explain the reduced pregnancy rates with a thin endometrium as described in other studies. In our study only 1/6 (16.7%) of the women with an endometrial thickness of 6 mm and 4/21 (19.0%) with an endometrial thickness of 7 mm had undergone a curettage and none an uterine radiation. Accordingly a curettage could be a reason for a thin endometrium is a few women but not in the majority.

On the other hand, the question arises of how relevant a reduced pregnancy rate with a thin endometrium really is. Of the 6 non-pregnant women in our study with an endometrial thickness of 6 mm, 3 women became pregnant later on. Thus, the clinical relevance of a thin endometrium without a recognizable cause such as multiple curettages etc., is questionable.

If a patient's endometrium is very thin, and if this may be a possible cause of infertility, the question of possible therapeutic options arises. Stimulation with estrogens can hardly be carried out in a spontaneous cycle, since high estrogen concentrations reduce FSH release and inhibit folliculogenesis and also impair endometrial function (16). Santamaria et al. (17), developed a treatment with bone marrow-derived stem cells, which seems to increase the chances of pregnancy in refractory Asherman's syndrome and endometrial atrophy. Whether such a complex and still experimental therapy is useful in cases with physiologically thin endometrium is questionable, since in such cases the endometrium is thinner but presumably functionally intact.

The differences in studies regarding the effect of a thick endometrium on the pregnancy rate are contradictory. In the gonadotrophin-stimulated IVF studies, a thick endometrium appears to be associated with a higher pregnancy rate (5–7). However, such a dependence could not be demonstrated in hormone-stimulated IUI therapies (8). We even found a tendency toward a reduced pregnancy rate. However, it needs to be noted that this finding is based on a statistical model which only provides a very vague tendency toward a lower pregnancy rate with very thick endometrium. Furthermore, the thickness which leads to decreased pregnancy rates cannot be defined.

The differences of the studies are barely explainable. It possibly concerns physiologically different endometrial function and IVF activity states, which do not allow a comparison of the different treatments as the endometrium is likely to be more proliferated and oedematous with gonadotrophin stimulation. It is also possible that the differences are due to the low number of patients in our study which can be considered a weakness of our study. Since the inclusion and exclusion criteria were very strict to be able to examine a patient population as homogeneously as possible, the patient numbers are limited.

In conclusion, the study confirmed that thin endometrium is also associated with lower pregnancy rates in unstimulated cycles. Therefore, thin endometrium should be regarded as an independent prognostic factor for achieving a pregnancy. However, as the pregnancy rate in women with thin endometrium is not zero but only reduced, thin endometrium should not be regarded as an infertility but rather as a fertility-reducing factor.

## Author Contributions

MvW designed the study, analyzed the data, and prepared the manuscript. MvW, MF, VM, PS, and AK collected data. MF prepared the data. MR and GG performed the statistics. All authors contributed to the data collecting, interpretation of the results, and the revision of the final manuscript.

### Conflict of Interest Statement

GG has received consultant fees from MSD, Merck Serono, Glycotope, Ferring, IBSA, VitroLife, Finox, ReprodWissen GmBH, and TEVA GmBH, ZIVA, Abbott, NMC Healthcare; has received speaker fees from Merck Serono, MSD, IBSA, VitroLife, ReprodWissen GmBH and Abbott. The remaining authors declare that the research was conducted in the absence of any commercial or financial relationships that could be construed as a potential conflict of interest.

## References

[1] Cakmak H Taylor HS. Implantation failure: molecular mechanisms and clinical treatment. Hum Reprod Update (2011) 17:242–53. 10.1093/humupd/dmq037 20729534 PMC3039220

[2] Singh N Bahadur A Mittal S Malhotra N Bhatt A. Predictive value of endometrial thickness, pattern and sub-endometrial blood flows on the day of hCG by 2D doppler in *in vitro* fertilization cycles: a prospective clinical study from a tertiary care unit. J Hum Reprod Sci. (2011) 4:29–33. 10.4103/0974-1208.82357 21772737 PMC3136066

[3] Kasius A Smit JG Torrance HL Eijkemans MJ Mol BW Opmeer BC. Endometrial thickness and pregnancy rates after IVF: a systematic review and meta-analysis. Hum Reprod Update (2014) 20:530–41. 10.1093/humupd/dmu011 24664156

[4] Weissman A Gotlieb L Casper RF. The detrimental effect of increased endometrial thickness on implantation and pregnancy rates and outcome in an *in vitro* fertilization program. Fertil Steril. (1999) 71:147–9. 10.1016/S0015-0282(98)00413-0 9935132

[5] Wu Y Gao X Lu X Xi J Jiang S Sun Y. Endometrial thickness affects the outcome of *in vitro* fertilization and embryo transfer in normal responders after GnRH antagonist administration. Reprod Biol Endocrinol. (2014) 12:96. 10.1186/1477-7827-12-96 25296555 PMC4197319

[6] Yuan X Saravelos SH Wang Q Xu Y Li TC Zhou C. Endometrial thickness as a predictor of pregnancy outcomes in 10787 fresh IVF-ICSI cycles. Reprod Biomed Online (2016) 33:197–205. 10.1016/j.rbmo.2016.05.002 27238372

[7] Ma NZ Chen L Dai W Bu ZQ Hu LL Sun YP. Influence of endometrial thickness on treatment outcomes following *in vitro* fertilization/intracytoplasmic sperm injection. Reprod Biol Endocrinol. (2017) 15:5. 10.1186/s12958-016-0222-5 28056983 PMC5216548

[8] Weiss NS van Vliet MN Limpens J Hompes PGA Lambalk CB. Endometrial thickness in women undergoing IUI with ovarian stimulation. How thick is too thin? A systematic review and meta-analysis. Hum Reprod. (2017) 32:1009–18. 10.1093/humrep/dex035 28333207

[9] von Wolff M Hua YZ Santi A Ocon E Weiss B. Follicle flushing in monofollicular *in vitro* fertilization almost doubles the number of transferable embryos. Acta Obstet Gynecol Scand. (2013) 92:346–8. 10.1111/aogs.12054 23194031 PMC3596803

[10] van Loendersloot LL van Wely M Limpens J Bossuyt PM Repping S van der Veen F. Predictive factors in *in vitro* fertilization (IVF): a systematic review and meta-analysis. Hum Reprod Update (2010) 16:577–89. 10.1093/humupd/dmq015 20581128

[11] Panidis D Tziomalos K Papadakis E Vosnakis C Betsas G Tsourdi E. Uterine volume and endometrial thickness in the early follicular phase in patients with polycystic ovary syndrome. Endocr Pract. (2014) 20:540–7. 10.4158/EP13058.OR 24325993

[12] Groenewould ER Ben J Al-Oraiby A Brinkhuis EA Broekmans FJM de Bruin JP Influence of endometrial thickness on pregnancy rates in modified natural cycle frozen-thawed embryo transfer. Acta Obstet Gynecol Scand. (2018) 97:808–15. 10.1111/aogs.13349 29582411

[13] Casper RF. It's time to pay attention to the endometrium. Fertil Steril. (2011) 96:519–21. 10.1016/j.fertnstert.2011.07.1096 21880272

[14] Azumaguchi A Henmi H Ohnishi H Endo T Saito T. Role of dilatation and curettage performed for spontaneous or induced abortion in the etiology of endometrial thinning. J Obstet Gynaecol Res. (2017) 43:523–9. 10.1111/jog.13254 28127830

[15] Critchley HO Bath LE Wallace WH. Radiation damage to the uterus – review of the effects of treatment of childhood cancer. Hum Fertil. (2002) 5:61–6. 10.1080/1464727022000198942 12082209

[16] Horcajadas JA Mínguez P Dopazo J Esteban FJ Domínguez F Giudice LC. Controlled ovarian stimulation induces a functional genomic delay of the endometrium with potential clinical implications. J Clin Endocrinol Metab. (2008) 93:4500–10. 10.1210/jc.2008-0588 18697870

[17] Santamaria X Cabanillas S Cervelló I Arbona C Raga F Ferro J. Autologous cell therapy with CD133+ bone marrow-derived stem cells for refractory Asherman's syndrome and endometrial atrophy: a pilot cohort study. Hum Reprod. (2016) 31:1087–96. 10.1093/humrep/dew042 27005892

